# CD14+CD16+ and CD14+CD163+ monocyte subpopulations in kidney allograft transplantation

**DOI:** 10.1186/1471-2172-15-4

**Published:** 2014-02-06

**Authors:** Alena Sekerkova, Eva Krepsova, Eva Brabcova, Janka Slatinska, Ondrej Viklicky, Vera Lanska, Ilja Striz

**Affiliations:** 1 Department of Clinical and Transplant Immunology, Institute for Clinical and Experimental Medicine, Videnska 1958/9, Prague 4 140 21, Czech Republic; 2 Transplant Laboratory, Institute for Clinical and Experimental Medicine, Prague, Czech Republic; 3 Department of Nephrology, Transplant Center, Institute for Clinical and Experimental Medicine, Prague, Czech Republic; 4 Department of Statistics, Institute for Clinical and Experimental Medicine, Prague, Czech Republic

**Keywords:** CD14+CD16+, CD14+CD163+, Kidney, Monocytes, Subpopulations, Transplantation

## Abstract

**Background:**

Monocytes represent a heterogeneous population of cells subdivided according to the expression level of membrane antigens. A pro-inflammatory (intermediate/nonclassical) subpopulation of monocytes is defined by expression of CD16. CD163 seems to be characteristically preferentially expressed by immunosuppressive monocytes. The aim of our study was to evaluate the distribution of monocyte subpopulations in 71 patients with kidney allograft transplantation.

**Results:**

The phenotype was evaluated by flow cytometry in defined time points. The proportions of peripheral CD14+CD16+ monocytes were downregulated immediately after the kidney transplantation and basiliximab treatment partially attenuated this trend. The transient downregulation of the CD14+CD16+ subpopulation was adjusted to basal values in two months. The proportions of CD14+CD163+ monocytes were transiently upregulated early after the kidney transplantation and remained higher during the first month in most patients. In ATG treated patients, the expansion of CD14+CD163+ monocytes was delayed but their upregulation lasted longer. In vitro data showed the direct effect of ATG and methylprednisolone on expression of CD16 and CD163 molecules while basiliximab did not affect the phenotype of cultured monocytes.

**Conclusions:**

We assume from our data that kidney allograft transplantation is associated with modulation of monocyte subpopulations (CD14+CD16+ and CD14+CD163+) partially affected by an immunosuppressive regime used.

## Background

In kidney transplantation, monocytic infiltration of the graft plays a key role in renal dysfunction [1] and their cytokines are actively involved in the process of acute rejection [2]. Peripheral blood monocytes represent highly efficient effector cells of innate immunity subdivided into different subpopulations by the expression level of membrane antigens CD14 (a receptor for bacterial LPS) and CD16 (Fc gamma RIII). These classical monocytes are characterized by a very high expression of CD14 and the absence of CD16 on their surface. Nevertheless, a subpopulation of monocytes does exist with lower CD14 expression and detectable CD16 molecule on a membrane [3]. These CD14+CD16+ intermediate/nonclassical monocytes represent up to 15% of peripheral blood monocytes but their numbers may be increased in patients with bacterial sepsis, TB or HIV infections [4]. These monocytes are smaller [5] and can be distinguished from CD14+CD16- monocytes by high HLA-DR and CD43 expression [6]. The subpopulations of monocytes also differ in the expression of chemokine receptors [7] and some of the functional activities. CD14+CD16+ monocytes are high producers of proinflammatory cytokine TNF alpha with limited capability to release anti-inflammatory IL-10 [8]. High expression of HLA-DR antigens might be one of the factors responsible for better antigen-presenting capacity of CD14+CD16+ monocytes [9]. In consideration of these properties, CD14+CD16+ cells should be considered as characteristic intermediate/nonclassical proinflammatory monocytes. The proportion of this monocytic subpopulation in the peripheral blood may be increased also in non-infectious inflammatory disorders such as Crohn’s disease [10], rheumatoid arthritis [11], bronchial asthma or sarcoidosis [12]. Furthermore, CD14+CD16+ monocytes are extensively studied with respect to the pathophysiology of atherosclerosis [13,14] including kidney transplant patients [15]. In contrast to these intermediate/nonclassical CD14+CD16+ monocytes, CD163 expression seems to be a marker of monocyte subset downregulating immune responses. This scavenger molecule for hemoglobin-haptoglobin complexes [16] functions also as a receptor for cytokine TWEAK [17] and some bacteria [18] and its expression is upregulated in response to glucocorticoids [19]. CD163 positive monocytes and macrophages (designed as M2 subset) are known to produce cytokine IL-10 suppressing effector immune mechanisms [20]. Among other myeloid antigens, the CD36 known to be upregulated during monocyte extravasation [21], and CD74, a receptor for macrophage migration inhibitory factor [22], represent additional markers of potential interest. In this respect, the aim of our prospective observational study was to monitore changes of peripheral monocyte subpopulations in early phases of kidney allograft transplantation with regard to different modes of induction immunosuppressive therapy.

## Methods

### Patients

In total, 20 healthy control and 71 patients who underwent renal transplantation from a deceased donor were enrolled in the study. Healthy controls were volunteers (age between 25–50 years) with no clinical symptoms, with no significant clinical diagnosis. None of the enrolled volunteers had transplantation in the past.

All patients were treated by a triple maintenance therapy consisting of calcineurin inhibitor (CNI, either tacrolimus or cyclosporine A), mycophenolate mofetil (MMF) and corticosteroids, with or without induction therapy. MMF and steroid therapy was started at day 0, CNI was given at day 0 or 1 of transplantation. Patients with panel reactive antibodies (PRA) >50% received 1–1.5 g/kg/day of rATG (Thymoglobulin®, Genzyme Corporation, Cambridge, MA, USA; n = 28) in 2 to 7 doses during the first postoperative week with respect to depletive effect. Patients with PRA 20-50% or those who received a kidney from extended criteria donor were treated by 20 mg of basiliximab (Simulect®, Novartis, Basel, Switzerland n = 18) on days 0 and 4. Low risk patients (PRA <20%) received no induction therapy (control group, n = 25).

Except for a difference in the frequency of retransplantation, mean percentage of panel reactive antibodies (PRA), and donor age, the clinical characteristics did not differ significantly between the three groups (Table 1). Peripheral blood samples were collected at following time-points: before transplantation and 7, 14, 21, 28, 60, 90 days and 6, 12 months post-transplantation. Serum creatinine (SCr), incidence of delayed graft function (DGF), estimated glomerular filtration rate (eGFR, calculated using MDRD_1_ formula), proteinuria and incidence of rejection during 3 months after transplantation and at 3-month protocol biopsy were recorded in patients. All patients gave their informed consent and signed their agreement with each biopsy performed. The study protocol was approved by the Ethics Committee of the Institute for Clinical and Experimental Medicine (No.:608-08-10).

**Table 1:** Demographic characteristics at the time of transplantation

	**No induction**		**rATG**		**Basiliximab**		**P-value**
Number	25		28		18		
Gender (male/female)	14/11		19/9		9/9		n.s.^b^
Recipient age (years)	57,9	[27.7; 73.3]	52,9	[21.5; 78.6]	52,9	[25.6; 67.1]	n.s.^d^
Donor age (years)	52.0	[16.0; 68.0]	46.5	[22.0; 74.0]	63.5	[46.0; 75.0]	<0.001^d,e^
^a^HLA MM	3	[1; 6]		[2; 5]		[1; 5]	n.s.^d^
1st/2nd and 3rd T x R (n)	25/0		12/16		18/0		<0.0001^b^
^a^PRA (%)	4	[0; 36]	64	[0; 96]	7	[0; 44]	<0.0001^d,g^
^a^CNI (TAC/CsA)(n)	21/4		28/0		18/0		n.s.^b^
^a^CIT (hours)	15.2	[11.0; 20.7]	15.9	[7.7; 22.8]	17.6	[12.4; 21.0]	n.s.^d^
Dialysis time (years)	2.0	[0.2; 9.42]	2.2	[0.5; 6.37]	2.1	[0; 4.9]	n.s.^d^
**Cause of renal failure**							n.s.
Primary GN	10		10		5		
Hereditary diseases	4		6		6		
Diabetic or ischaemic nephropathy	8		2		5		
^a^TIN	2		3		1		
^a^ANCA vasculitis or lupus nephritis	0		4		0		
Other causes	1		3		1		

^a^ANCA, anti-neutrophil cytoplasmic antibodies; CIT, cold ischaemic time; CNI, calcineurin inhibitor; CsA, cyclosporine A; GN, glomerulonephritis; HLA MM, HLA mismatch; PRA, historical panel reactive antibodies, measured every 3 months before transplantation, the highest number was considered in each patient; TAC, tacrolimus; TIN, tubulointerstitial nephritis; T 3, renal transplantation.

^b^Chi square test P-value.

^d^Kruskal–Wallis test P value.

^e^Dunn’s multiple comparison test: significant difference between the basiliximab group and the rATG or controls.

^g^Dunn’s multiple comparison test: significantp difference between rATG and the no induction or basiliximab groups.

All patients underwent renal graft biopsy upon clinical presumption of acute rejection such as insufficient decline or sudden rise of serum creatinine. Furthermore, patients underwent the protocol biopsy at three months after transplantation. Biopsy-proven acute rejection was diagnosed histologically according to the Banff ’05 classification [23]. Borderline changes and T cell-mediated rejection (TCMR) grade I and IIA were treated by 1.5-2 g of methylprednisolone, TCMR grades IIB, III and steroid-resistant TCMR by rATG (10 dose; 2 mg/kg 1st day and 1 mg/kg 2nd-10th day of treatment) and antibody-mediated rejection (AMR) by plasma exchange and intravenous immunuglobulin alternately during 10 days.

Three patients with no induction who obtained rATG as a treatment of severe acute rejection (first dose: day 12, 8 and 8) were excluded from group comparison statistics of flow cytometry data.

### Flow cytometry

Peripheral blood mononuclear cells (100 ul, approx. 1*10^6^) were labeled with fluorochrome-conjugated (PE – phycoerythrin, PC7 – phycoerythrin-cyanine 7, PC5 – phycoerythrin-cyanine 5, FITC – fluorescein) monoclonal antibodies resuspended in PBS-BSA buffer for 20′ at room temperature (RT) in the dark. The following antibodies were used: CD36-FITC, CD14-PC7, CD16-ECD (Beckman Coulter, Brea, CA, USA), CD74-Alexa Fluor 647, CD163 (clone GHI61)-PE, CD163 (clone RM3/1)-PE (BioLegend). Fresh blood (100 ul) with monoclonal antibodies were incubated 20′ in the room temperature in the dark. Samples were lysed with 0.5 ml lysing solution OptilyseC (Beckman Coulter, Brea, CA, USA) 10′ the room temperature in the dark. Lysing reaction was stoped with 1 ml CellWash (optimized PBS) (Beckton Dickinson Bioscience, Benelux, Belgium). Samples were measured on a FC 500 flow cytometer (Beckman Coulter, Brea, CA, USA) and analyzed using CxP software and Kaluza software (Beckman Coulter, Brea, CA, USA). Gating strategy: monocytes were gated by side scatter and CD14 expression. Subsequently CD 16, CD36, CD74 and CD163 were measured on the cell surfaces. A flow cytometry analysis was performed with at least 100 events in the gate. The absolute number of monocyte subpopulations are calculated from the absolute number of leukocytes and are shown as number of cells * 10^6^/l. Absolute number of leukocytes was measured by hematology analyzer Sysmex (Sysmex Corporation, Japan).

### “In vitro” cultures of peripheral blood mononuclear cells ( PBMCs)

Three healthy volunteers (between 30–55 years) with no clinical symptoms, with no significant clinical diagnosis and without transplantation in the past were selected for in vitro experiments. Fresh blood (50 ml) was collected from each individual into a tube containing EDTA.

PBMCs were purified using standard Ficoll-Paque gradient centrifugation. Briefly, 3 ml of Ficoll-Paque gradient was pipetted into a 15-ml centrifuge tube. The EDTA blood was diluted 1:1 in phosphate-buffered saline (PBS) and carefully layered over the Ficoll-Paque gradient (3 ml/tube). The tubes were centrifuged for 30 min at 580 × *g*. The cell interface layer was harvested carefully and the cells were washed once in PBS (for 10 min at 180 × *g*) and then once in RPMI-1640 medium (Sigma-Aldrich, St. Louis, MO, USA) supplemented with 10% heat inactivated fetal calf serum (FCS), L-glutamin, penicillin and streptomycin (Sigma-Aldrich), (for 10 min at 180 × *g*). Cells were harvested from the surface by using Trypsin-EDTA (Sigma-Aldrich, St. Louis, MO, USA).

Isolated cells were cultured in duplicates in 24-well tissue culture plates (Costar, Corning, NY, USA) under a 5% CO_2_ atmosphere at 37° in RPMI-1640 supplemented with ATG (1, 10 and 100 ug/ml), basiliximab (1, 10, 100 and 1000 ng/ml) or methylprednisolon (10, 100, 1000 ug/ml). Non-stimulated cells were used as a negative control. The proportions of CD14+CD16+ and CD14+CD163+ monocytes were evaluated in duplicates by flow cytometry at times 0;1;3;6;24;48 and 72 hours after stimulation. Cell viability was evaluated using a Vi-Cell analyzer (Beckman Coulter, Brea, CA, USA). In ATG-stimulated cells, 72 h culture affected the viability of monocytes and data were not analyzed at this time point.

### Statistics

Statistical analyses were performed by GraphPad Prism 5 software (GraphPad Software, La Jolla, CA, USA) and BMDP PC-90 statistical software (BMDP Statistical Software Inc., Los Angeles, CA, USA). Based on the distribution of the data (Shapiro-Wilk’s test for normality), we performed parametric or nonparametric (Mann–Whitney, Kruskal-Wallis) testing. For analysis of categorical variables, the chi-square test was used. Data are showed as median [min; max] according to the distribution. Analysis of data from flow cytometry was performed by ANOVA with repeated measures, comparison within groups between two time points by contrasts. Dynamics of the parametres was fitted by quadratic regression and the estimated coefficients were compared by z-statistcs.

## Results

### Patient and graft survival

From a total of 71 patients, three patients underwent graft nephrectomy (Day 4, 20 and 58 after transplantation) for renal vein thrombosis, acute hemorrhage after biopsy or primary non-functional graft, respectively; 2 patients died on Days 56 and 80 of sudden death and pulmonal artery embolism, respectively. Two patients withdrew their informed consent to participate in the study at Days 14 and 60.

### Graft function

The three patient groups examined did not differ in the incidence rates of DGF: controls: 8/25 (32%); the rATG group: 7/28 (25%) and the basiliximab group: 9/18 (50%). No significant differences in the levels of SCr, eGFR and proteinuria were observed, respectively, between treatment groups 90 days post-transplantation: controls, 127 [61; 314] μmol/L, 0.76 [0.19; 1.46] mL/s/1.73 m2 and 0.22 [0.07; 2.18] g/24 h; rATG, 120 [57; 239] μmol/L, 0.78 [0.29; 1.57] mL/s/1.73 m2 and 0.18 [0.10; 2.15] g/24 h and basiliximab, 153 [79; 278] μmol/L, 0.59 [0.28; 1.10] mL/s/1.73 m2 and 0.28 [0.07; 0.49] g/24 h.

### Graft rejection

Acute rejection occurred during the first 3 months in 4/24 (16.7%) control group patients, 3/28 (10.7%) rATG patients and in 2/18 (11.1%) basiliximab-treated patients (P > 0.05). There were another five patients with borderline changes in the control group and seven were treated with basiliximab. In patients without induction therapy, two patients suffered from early acute TCMR, one patient from acute AMR and one patient had a combined type of rejection. In patients with rATG induction, three patients suffered from AMR and in those with basiliximab, one patient suffered from early acute TCMR and one from combined acute TCMR and AMR.

### Peripheral blood CD14+CD16+ monocytes after kidney allograft transplantation

**Figure 1:**
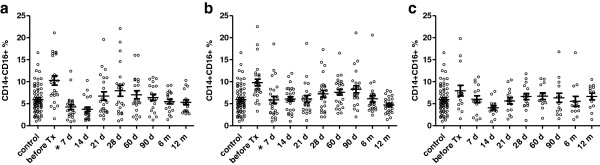
**Percentage of peripheral blood CD14**+**CD16+ monocytes after kidney allograft transplantation.** The co-expression of CD14 and CD16 antigens on peripheral blood monocytes was evaluated by flow cytometry in three different groups of patients undergoing kidney allograft transplantation (**a**-without induction, **b**-thymoglobulin, **c**-basiliximab). In patients without the induction therapy (n = 25 ) and those treated with thymoglobulin (n = 28 ), the percentage of CD14+CD16+ monocytes decreased during the first two weeks after the transplantation as compared to only moderate changes observed in subjects treated with basiliximab (n = 18). Analysis of data from flow cytometry was performed by ANOVA with repeated measures and a comparison within groups between two time points by contrasts. *Statistically significant differences.

**Figure 2:**
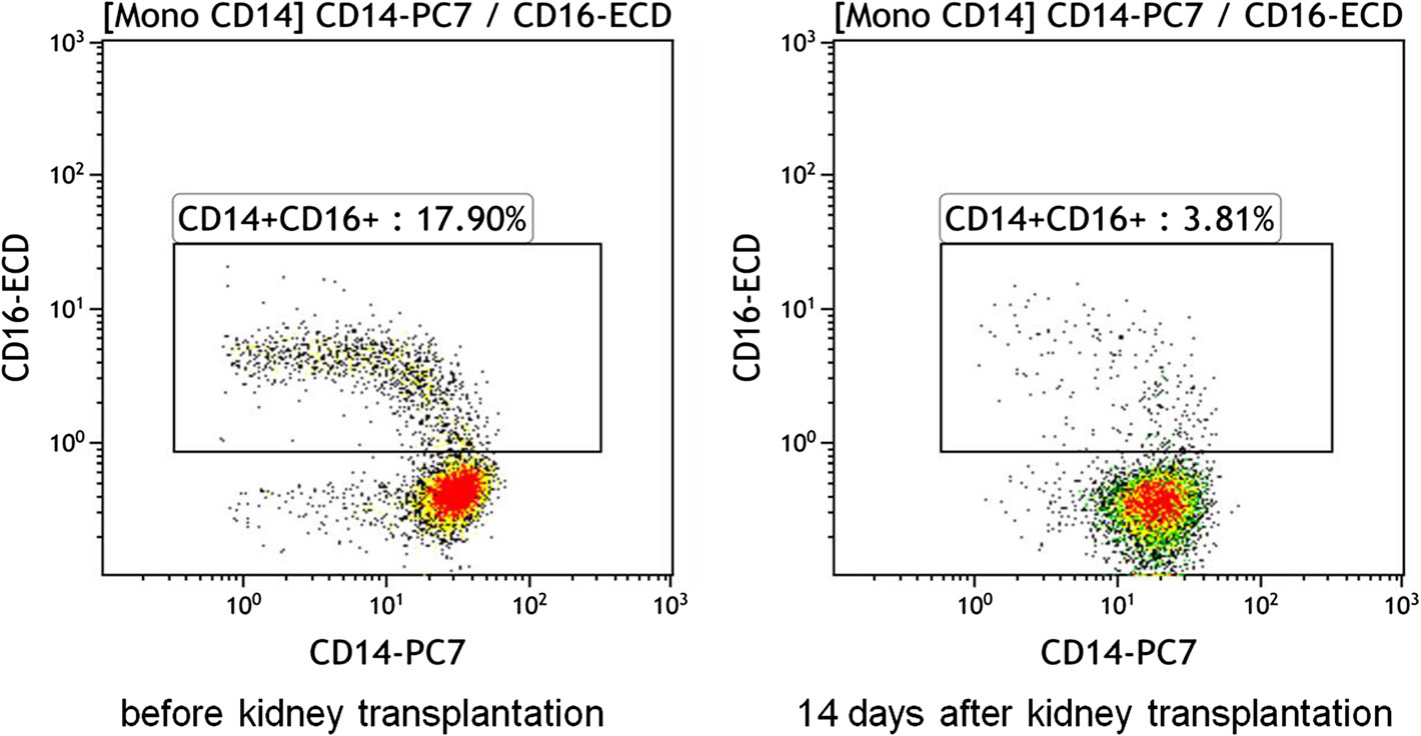
**Downregulation of CD14**+**CD16+ monocyte subpopulation after the kidney transplantation.** The flow cytometry plot expresses the data of one representative patient without the induction therapy. The number of CD14+CD16+ were strongly downregulated during the first week after the kidney transplantation.

The percentage of peripheral CD14+CD16+ monocytes was found to be downregulated during 7 days after the kidney transplantation in the group of patients without the induction therapy (p < 0.0005) and those treated with thymoglobulin (p < 0.0002). In patients treated with anti-CD25 antibody basiliximab, the decrease in percentage of CD14+CD16+ reached the minimum after two weeks and the moderate changes did not reach statistical significance (p = ns) (Figures 1 and 2). In most patients, the transient downregulation of CD14+CD16+ subpopulation was almost adjusted in three months but did not reach the values obtained before the transplantation.

### Peripheral blood CD14+CD163+ monocytes after kidney allograft transplantation

**Figure 3:**
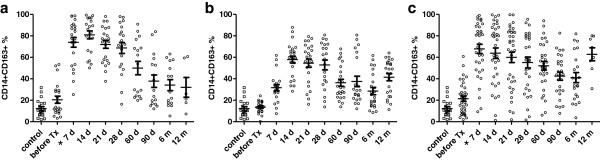
**Percentage of peripheral blood CD14**+**CD163+ monocytes after kidney allograft transplantation.** The presence of CD14+ CD163+ monocytes in peripheral blood was evaluated by flow cytometry during the first year after the kidney allograft transplantation. In patients without the induction therapy **a** (n = 25 ) and those treated with basiliximab **c** (n = 18 ), the percentage of CD14+CD163+ monocytes dramatically increased during the first week after the transplantation in contrast to subjects treated with thymoglobuline **b** (n = 24). Analysis of data from flow cytometry was performed by ANOVA with repeated measures and a comparison within groups between two time points by contrasts. *Statistically significant differences.

**Figure 4:**
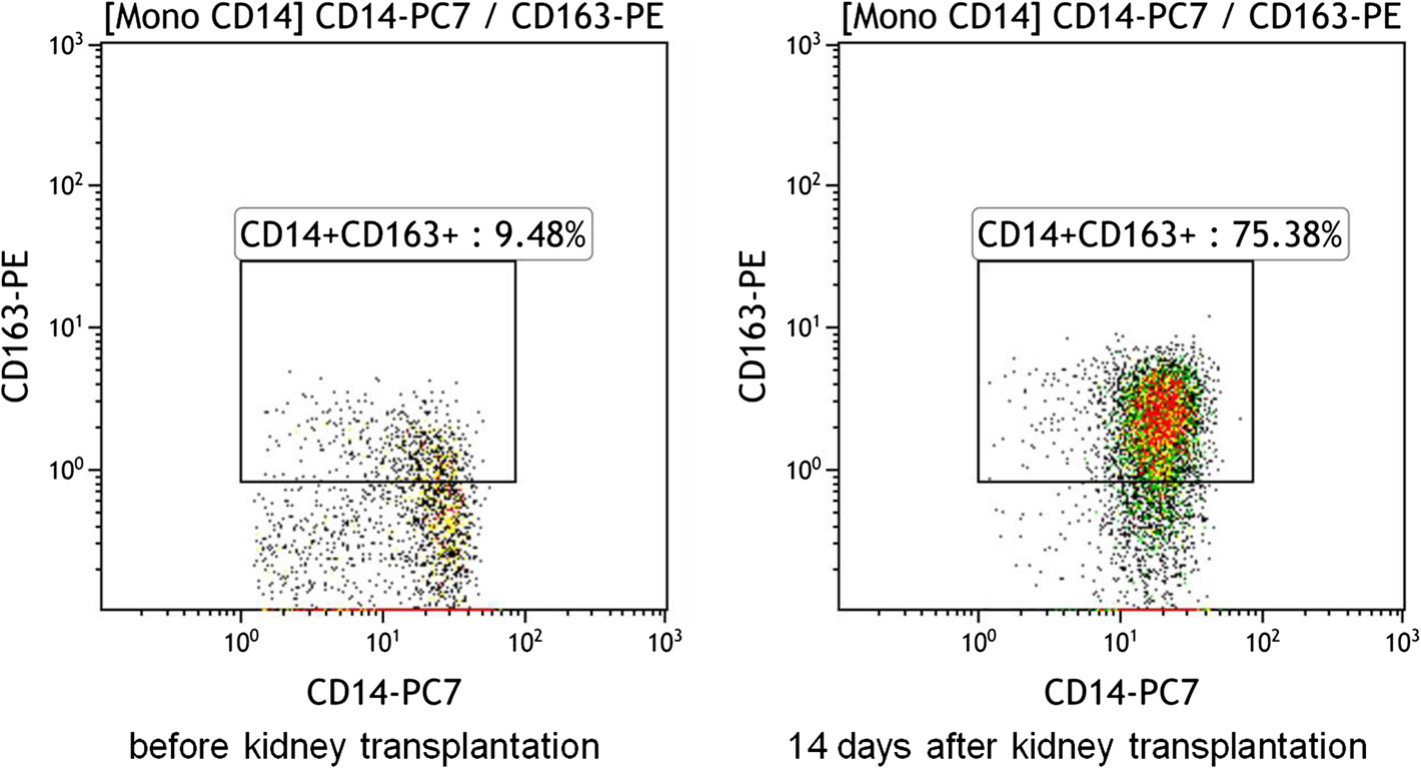
**Expansion of CD14**+**CD163+ monocytes following kidney transplantation.** The flow cytometry plot expresses the data of one patient with basiliximab induction therapy. The percentage of CD14+CD163+ monocytes was highly upregulated during the first week after the transplantation.

**Figure 5:**
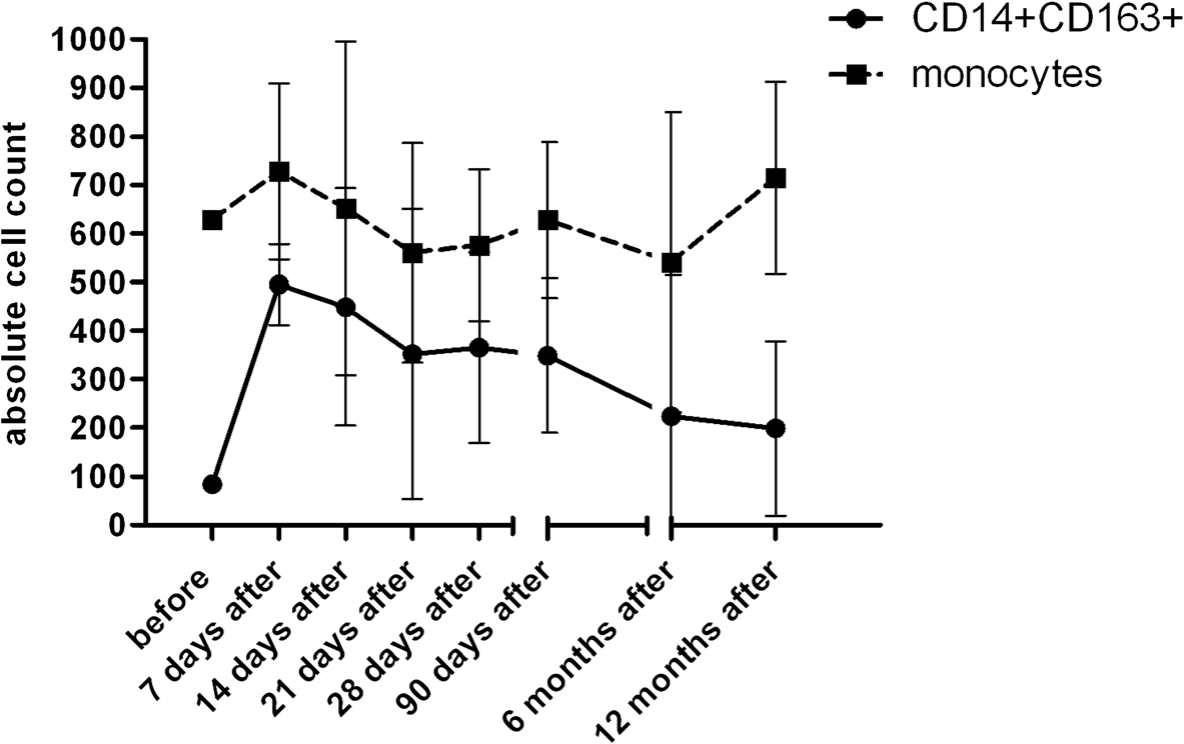
**Proportional changes of CD14**+**CD163+ subpopulation as compared to the whole population of peripheral blood monocytes.** The absolute numbers of peripheral blood monocytes and their CD14+CD163 subpopulation were monitored during the first year following kidney allograft transplantation (n = 71). The early massive upregulation of CD14+CD163+ subpopulation is in a contrast with only moderate changes observed in whole population of peripheral blood monocytes (absolute numbers of monocyte subpopulations are calculated from the total number of leukocytes and are expressed as number of cells *10^6^/l).

The percentage of peripheral CD14+CD163+ monocytes was found to be upregulated during 7 days after the kidney transplantation in the group of patients without the induction therapy (p < 0.0001) and those treated with basiliximab (p < 0.0001). In patients treated with thymoglobulin, the upregulation in percentage of CD14+CD163+ reach the maximum after two weeks (p < 0.001) (Figures 3 and 4). In most patients, the upregulation of CD14+CD163+ subpopulation was almost adjusted in three or six months but did not reach the values obtained before the transplantation. In the pooled group of kidney transplant recipients (n = 71), marked upregulation of CD14+CD163 subpopulation is in contrast with minimal changes in whole monocyte population (Figure 5).

### Peripheral blood CD14+CD16+ and CD14+CD163+ monocytes in patients with acute rejection of kidney allograft

**Figure 6:**
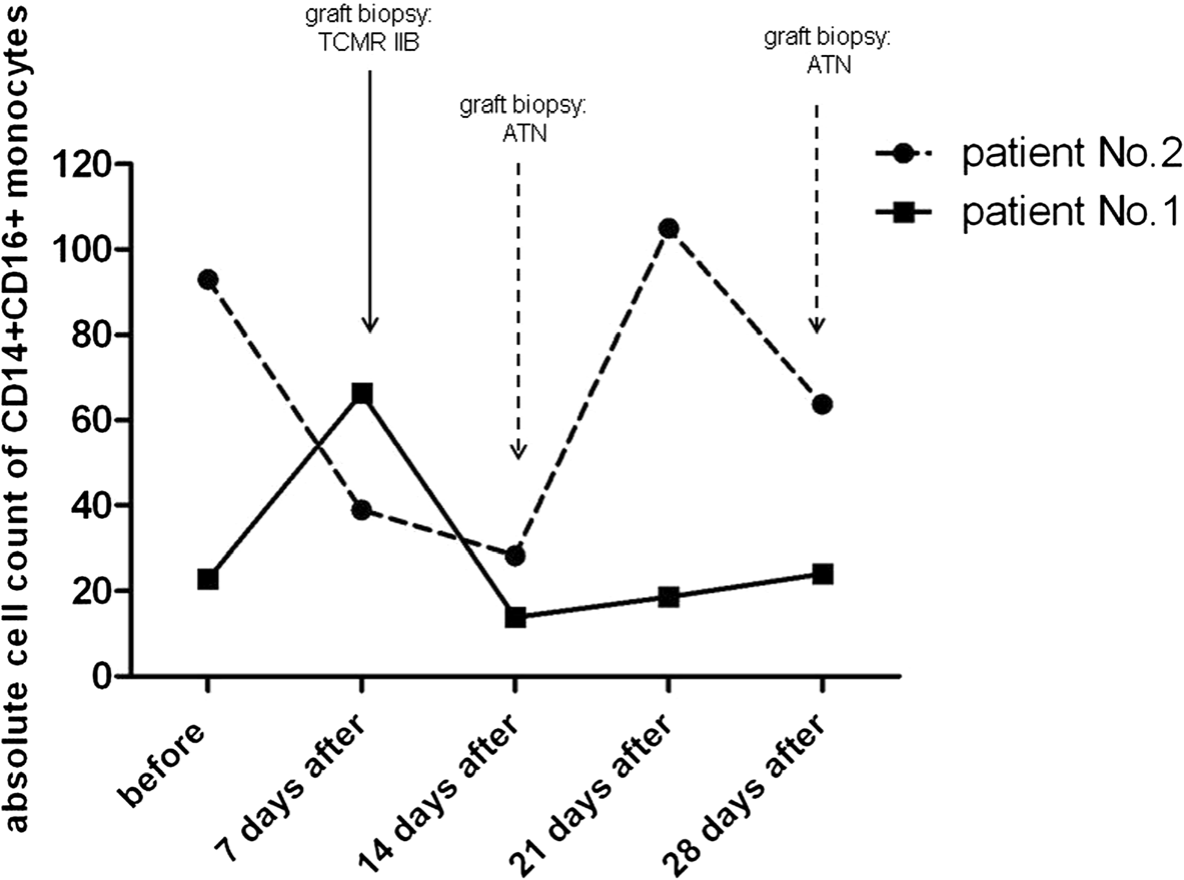
**Case reports of two patients with complicated outcome of kidney allograft transplantation associated with changes of CD14**+**CD16+ subpopulation.** Patient No.1 is a 56-year old male with delayed graft function and diagnosed acute cellular rejection IIB in early phase following kidney allograft transplantation, treated successfully with thymoglobulin. Patient N. 2 is a 51-year old male with C4d negative humoral rejection (FACSXM positive, presence of MICA antibodies) diagnosed one month after the transplantation. After changing of immunosuppression and subsequent IVIG therapy, the kidney functions became stable. In both cases, the clinical status of patients correlated with proportions of CD14+CD16+ monocytes. TCMR = T cell-mediated rejection, ATN = acute tubular necrosis (absolute numbers of monocyte subpopulations are calculated from the total number of leukocytes and are expressed as number of cells *10^6^/l).

Our preliminary data obtained from two patients undergoing acute rejection showed the trend to upregulation of CD14+CD16+ monocytes in these subjects as demonstrated in a case report of two study subjects (Figure 6).

### “In vitro” effect of immunosuppressives on phenotype of human monocytes

**Figure 7:**
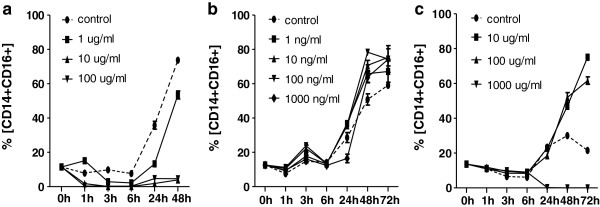
**In vitro effect of immunosuppressives on the percentage of CD14**+**CD16+ monocytes peripheral blood monocytes collected from a healthy donor were cultured in the presence of rATG, basiliximab and methylprednisolone.** Changes of CD16 expression on the surface CD14-positive monocytes were measured at following time points: 0, 1, 3, 6, 24, 48 and 72 h. Incubation with rATG **(a)** downregulated the proportion of CD14+CD16+ monocytes already after one hour. Basiliximab **(b)** had no effect. Methylprednisolone **(c)** upregulated the percentage of CD14+CD16+ subpopulation. Dynamics of the parameters was fitted by quadratic regression and the estimated coefficients were compared by z-statistcs.

In order to assess the direct effect of immunosuppressives, the proportions of monocyte subpopulations CD14+CD16+ and CD14+CD163+ were evaluated in cultured PBMC exposed to rATG, basiliximab, and methylprednisolone. Exposition of PBMC to rATG (Figure 7a) reduced the percentage of CD14+CD16+ monocytes in 1 hour and the decline continued until 48 hours. In contrast, percentage of CD14+CD16+ monocytes in control samples gradually grew up to 75% from initial 10% of cells (p < 0.05). The data suggest that rATG is effective already at very low concentrations. In contrast, basiliximab does not affect the proportions of CD14+CD16+ monocytes at all (Figure 7b). Methylprednisolone (Figure 7c) upregulated the percentage of CD14+CD16+ monocytes after 48 hours (p < 0.05) compared to the control.

**Figure 8:**
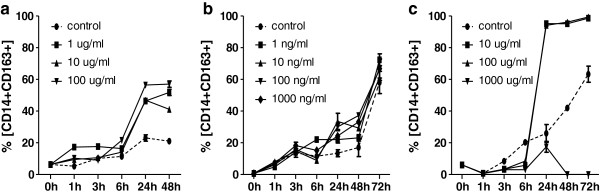
**In vitro effect of immunosuppressives on the percentage of CD14**+**CD163+ monocytes peripheral blood monocytes collected from a healthy donor were cultured in the presence of rATG, basiliximab and methylprednisolone.** Membrane expression of CD163 on cultured monocytes was measured at following time points: 0, 1, 3, 6, 24, 48 and 72 h. Both the rATG **(a)** and methylprednisolone **(b)** upregulated the proportion of CD14+CD163+ monocytes while basiliximab **(c)** had no effect. Dynamics of the parameters was fitted by quadratic regression and the estimated coefficients were compared by z-statistcs.

Monocytes incubated with rATG (Figure 8a) upregulated the expression of CD163+ after 6 hours and the proportion of CD14+CD163+ monocytes increased from 6% at the time point 0 h to 50% at the time point 48 h (p < 0.01) . In control cultures of PBMCs, the percentage of CD14+CD163+ monocytes reach the maximum of 20% after 48 hours (p < 0.05). Basiliximab at the all concentrations and time points did not modulate the development of CD14+CD163+ monocytes (Figure 8b). Methylprednisolon (10 and 100 μg) has been shown to be the strongest inducer of CD14+CD163+ monocytes and upregulated their proportions from 6% to 90% in the first 6 hours (p < 0.05) (Figure 8c). Concentrations higher than 1000 ug/ml were found to be toxic, the results of cell viability tests are shown in the Table 2.

**Table 2:** **Viability of the cells (“ *in vitro *” experiments)**

	**rATG**					
**Hour**		**Control**	**1 µg/ml**	**10 µg/ml**	**100 µg/ml**	
	0	94,1 ± 1,0	96,1 ± 1,3	96,3 ± 1,8	95,1 ± 2,2	
	24	97,4 ± 1,8	95,9 ± 1,8	94,2 ± 3,2	93,2 ± 1,9	
	48	93,2 ± 2,6	94,6 ± 2,1	95,6 ± 1,9	92,7 ± 1,3	
	72	97,3 ± 1,4	96,1 ± 2,5	94,8 ± 2,1	95,2 ± 1,2	
	**Basiliximab**					
**Hour**		**Control**	**1 ng/ml**	**10 ng/ml**	**100 ng/ml**	**1000 ng/ml**
	0	96,1 ± 1,1	95,8 ± 1,9	95,3 ± 1,6	93,6 ± 0,8	93,5 ± 1,8
	24	97,3 ± 2,1	92,1 ± 2,1	96,2 ± 1,1	94,6 ± 1,2	93,8 ± 2,5
	48	97,2 ± 1,8	96,4 ± 2,2	98,1 ± 0,5	93,8 ± 2,1	95,1 ± 1,8
	72	96,3 ± 2,4	93,9 ± 1,9	95,6 ± 2,1	94,3 ± 3,1	96,2 ± 1,3
	**Methylprednisolone**					
**Hour**		**Control**	**10 µg/ml**	**100 µg/ml**	**1000 µg/ml**	
	0	95,1 ± 1,3	95,1 ± 1,2	96,9 ± 2,1	84,2 ± 3,8	
	24	96,9 ± 2,5	95,8 ± 1,8	96,9 ± 1,8	33,4 ± 8,9	
	48	96,7 ± 1,3	96,2 ± 2,2	95,1 ± 2,1	38,1 ± 10,3	
	72	95,6 ± 2,1	97,0 ± 1,9	95,3 ± 2,3	31,1 ± 10,2	

Viability of the cells was measured on the bases of penetration of trypan blue into the cells.

### Peripheral blood monocyte expression of CD36 and CD74 after kidney allograft transplantation

Constitutive CD36 expression was found in more than 98% of monocytes without any dynamic changes during the post-transplantation period. Similarly, a high density CD74 expression was present in all peripheral blood monocytes without any changes. Proportions of peripheral blood monocytes in the frame of whole blood count were not affected during the 12 months period after the kidney allograft transplantation (not shown).

## Discussion

Our data suggest that kidney allograft transplantation is associated with evident shifts in proportions of specific monocyte subpopulations CD14+CD16+ and CD14+CD163+ and these changes are partially affected by the immunosuppressive regime used. During the first week after the transplantation, the proportion of intermediate/nonclassical CD14+CD16+ monocytes is downregulated which might be due to their trafficking to the graft or by their selective deletion. Obviously, no bioptical data to prove the presence of CD14+CD16 monocytes in the graft immediately after the surgery were obtainable from ethical reasons. Nonetheless, selective deletion of CD14+CD16+ monocytes seems to us more relevant since recent data showing that these monocytes are senescent [24] also indirectly suggest that they might be more sensitive to the aggressive environment of a “cytokine storm” associated with the ischemia/reperfusion injury. The initial values of CD14+CD16+ monocytes before the kidney transplantation were higher as compared to values in healthy subjects reflecting the end stage kidney disease [14].

The effect of different treatment regimes was also observed. A statistically significant decrease of the CD14+CD16+ monocytes in the first week after transplantation was observed in patients without induction therapy and those treated with rATG. Patients treated with basiliximab achieved only moderate decrease after two weeks of treatment. In this respect, we also demonstrated the direct effect of rATG on the decrease of CD14+CD16+ monocytes in cultured PBMCs while basiliximab did not affect this monocytic subpopulation “in vitro”. The reason for the different cellular response may be due to different mechanism of action of rATG and basiliximab. Polyclonal rATG leads to an immediate targeted depletion of T lymphocytes together with evident effects on other immune cells while basiliximab binds to the α-chain of IL-2R (CD25) on the T lymphocyte surface, so that T lymphocytes are not able to bind to IL-2. This treatment affects mainly IL-2 and T lymphocyte mediated immune responses and probably does not directly affect monocytes [25].

Early upregulation of CD163 expression might be a result of prednisolone treatment as a part of standard immunosuppressive regimes since in vitro, the induction of this molecule on monocytes by glucocorticoids has been already documented [19] and our data confirmed early induction of CD163 expression on monocytes in response to methylprednisolone. In addition to increasing the number of monocytes expressing CD163, methylprednisolone also upregulated the density CD163 on the cells surface.

Surprisingly, the delayed effect of CD163 induction in thymoglobulin-treated patients was associated with its prolonged expression and might be regulated by other induced cells since it lasted for 3 months after the last treatment with this polyclonal antibody. The initial proportions of CD14+CD163+ monocytes did not differ from values of healthy subjects.

Simultaneously “in vivo”, we found a decreased number of total peripheral blood monocytes one week after transplantation in the patients group receiving rATG. The ability of rATG to induce apoptosis of monocytes has been demonstrated. It is assumed that monocyte depletion by rATG might contribute to the prevention of GVHD [26]. rATG effect on the monocytes function, including the expression of surfaces markers and production of cytokine spectrum, can be expected.

Furthermore, we have analyzed a potential relationship between the proportion of monocyte subpopulations and PRA values and have not found any correlations (data not shown). In few patients undergoing acute rejection of kidney allograft, the intermediate/nonclassical CD14+CD16+ monocytes are upregulated and their capacity to release cytokines like TNF alpha and IL-1 beta might be indirectly involved in downregulation of the CD14+CD163+ phenotype. In human monocytes, CD163 expression is known to be downregulated in vitro by IFN gamma [27], a cytokine associated with Th1 mediated rejection mechanisms. Our data did not support this pattern of regulation in kidney transplant patients.

With respect to standardization procedures, our experiments confirmed the differences in domain specific binding of anti-CD163 antibodies which are responsible for published discrepancies in CD163 monocyte positivities [28]. We tested two different clones (RM3/1 and GHI61) for the detection of CD163 but only GHI61 clone bound to the surface of blood monocytes in contrast to the epitope recognized by clone RM3/1 expressed exclusively on the surface of macrophages and completely absent in monocytes (data not shown).

## Conclusions

Both the initial phase of kidney transplantation immediately after the graft reperfusion and acute rejection process are associated with multiple immunological reactions and changes in the proportions of monocyte subpopulations might be one of the key factors. Estimating the number of peripheral intermediate CD14+CD16+ and “anti-inflammatory” CD14+CD163+ monocytes might be one of new potentially perspective laboratory parameters to evaluate immune responses in kidney transplant recipients. The induction of immunosuppressive CD14+CD163+ monocytes and downregulation of proinflammatory intermediate/nonclassical CD14+CD16+ monocytes might play a protective role in the early phase after kidney transplantation. On the other hand, we have not performed any functional tests to confirm immunosuppressive or proinflammatory properties of these cells in this study.

## Abbreviations

LPS: Lipopolysaccharide; TB: Tuberculosis; TNF alpha: Tumor necrosis factor- alpha; IL-10: Interleukin 10; CNI: Calcineurin inhibitor; MMF: Mycophenolate mofetil; PRA: Panel reactive antibodies; rATG: Thymoglobulin; SCr: Serum creatinine; DGF: Delayed graft function; eGFR: estimated glomerular filtration rate; TCMR: T cell-mediated rejection; AMR: Antibody-mediated rejection; PE: Phycoerythrin; PC7: Phycoerythrin-cyanine 7; PC5: Phycoerythrin-cyanine 5; FITC: Fluorescein isothiocyanate; IL-1 beta: Interleukin-1 beta; IFN gama: Interferon gamma.

## Competing interests

The author declares that they have no competing interests.

## Authors’ contributions

AS performed flow cytometry analysis, analyzed data, wrote the paper. EK collected and analyzed clinical data. EB performed flow cytometry analysis, analyzed data, made graphs. JS collected and analyzed clinical data. OV designed study, analyzed data. VL completed statistical analysis. IS designed study, analyzed data, wrote the paper. All authors read and approved the final manuscript.

## References

[B1] Girlanda R Kleiner DE Duan Z Ford EA Wright EC Mannon RB Kirk AD Monocyte infiltration and kidney allograft dysfunction during acute rejection Am J Transplant 2008 8 3 600 607 10.1111/j.1600-6143.2007.02109.x 18294156 PMC2813043

[B2] Hribova P Lacha J Kotsch K Volk HD Brabcova I Skibova J Vitko S Viklicky O Intrarenal cytokine and chemokine gene expression and kidney graft outcome Kidney Blood Press Res 2007 30 5 273 282 10.1159/000105134 17622765

[B3] Passlick B Flieger D Ziegler-Heitbrock HW Identification and characterization of a novel monocyte subpopulation in human peripheral blood Blood 1989 74 7 2527 2534 2478233

[B4] Ziegler-Heitbrock L The CD14+ CD16+ blood monocytes: their role in infection and inflammation J Leukoc Biol 2007 81 3 584 592 Epub 2006 Nov 2029 17135573 10.1189/jlb.0806510

[B5] Ziegler-Heitbrock HW Strobel M Fingerle G Schlunck T Pforte A Blumenstein M Haas JG Small (CD14+/CD16+) monocytes and regular monocytes in human blood Pathobiology 1991 59 3 127 130 10.1159/000163629 1715711

[B6] Komohara Y Hirahara J Horikawa T Kawamura K Kiyota E Sakashita N Araki N Takeya M AM-3K, an anti-macrophage antibody, recognizes CD163, a molecule associated with an anti-inflammatory macrophage phenotype J Histochem Cytochem 2006 54 7 763 771 Epub 2006 Mar 2003 10.1369/jhc.5A6871.2006 16517975

[B7] Weber C Belge KU von Hundelshausen P Draude G Steppich B Mack M Frankenberger M Weber KS Ziegler-Heitbrock HW Differential chemokine receptor expression and function in human monocyte subpopulations J Leukoc Biol 2000 67 5 699 704 10811011 10.1002/jlb.67.5.699

[B8] Belge KU Dayyani F Horelt A Siedlar M Frankenberger M Frankenberger B Espevik T Ziegler-Heitbrock L The proinflammatory CD14+CD16 + DR++ monocytes are a major source of TNF J Immunol 2002 168 7 3536 3542 11907116 10.4049/jimmunol.168.7.3536

[B9] Randolph GJ Jakubzick C Qu C Antigen presentation by monocytes and monocyte-derived cells Curr Opin Immunol 2008 20 1 52 60 Epub 2007 Dec 2021 10.1016/j.coi.2007.10.010 18160272 PMC2408874

[B10] Grip O Bredberg A Lindgren S Henriksson G Increased subpopulations of CD16(+) and CD56(+) blood monocytes in patients with active Crohn’s disease Inflamm Bowel Dis 2007 13 5 566 572 10.1002/ibd.20025 17260384

[B11] Kawanaka N Yamamura M Aita T Morita Y Okamoto A Kawashima M Iwahashi M Ueno A Ohmoto Y Makino H CD14+, CD16+ blood monocytes and joint inflammation in rheumatoid arthritis Arthritis Rheum 2002 46 10 2578 2586 10.1002/art.10545 12384915

[B12] Okamoto H Mizuno K Horio T Circulating CD14+ CD16+ monocytes are expanded in sarcoidosis patients J Dermatol 2003 30 7 503 509 12928539 10.1111/j.1346-8138.2003.tb00424.x

[B13] Waldo SW Li Y Buono C Zhao B Billings EM Chang J Kruth HS Heterogeneity of Human Macrophages in Culture and in Atherosclerotic Plaques Am J Pathol 2008 5 5 10.2353/ajpath.2008.070513 PMC2276432 18321997

[B14] Rogacev KS Seiler S Zawada AM Reichart B Herath E Roth D Ulrich C Fliser D Heine GH CD14++CD16+ monocytes and cardiovascular outcome in patients with chronic kidney disease Eur Heart J 2011 32 1 84 92 10.1093/eurheartj/ehq371 20943670

[B15] Ulrich C Heine GH Gerhart MK Kohler H Girndt M Proinflammatory CD14+CD16+ monocytes are associated with subclinical atherosclerosis in renal transplant patients Am J Transplant 2008 8 1 103 110 Epub 2007 Nov 2012 18021284 10.1111/j.1600-6143.2007.02035.x

[B16] Fabriek BO Dijkstra CD van den Berg TK The macrophage scavenger receptor CD163 Immunobiology 2005 210 2–4 153 160 16164022 10.1016/j.imbio.2005.05.010

[B17] Moreno JA Munoz-Garcia B Martin-Ventura JL Madrigal-Matute J Orbe J Paramo JA Ortega L Egido J Blanco-Colio LM The CD163-expressing macrophages recognize and internalize TWEAK: potential consequences in atherosclerosis Atherosclerosis 2009 207 1 103 110 10.1016/j.atherosclerosis.2009.04.033 19473660

[B18] Fabriek BO van Bruggen R Deng DM Ligtenberg AJ Nazmi K Schornagel K Vloet RP Dijkstra CD van den Berg TK The macrophage scavenger receptor CD163 functions as an innate immune sensor for bacteria Blood 2009 113 4 887 892 10.1182/blood-2008-07-167064 18849484

[B19] Maniecki MB Moller HJ Moestrup SK Moller BK CD163 positive subsets of blood dendritic cells: the scavenging macrophage receptors CD163 and CD91 are coexpressed on human dendritic cells and monocytes Immunobiology 2006 211 6–8 407 417 16920480 10.1016/j.imbio.2006.05.019

[B20] Mayer A Lee S Jung F Grutz G Lendlein A Hiebl B CD14+ CD163+ IL-10+ monocytes/macrophages: Pro-angiogenic and non pro-inflammatory isolation, enrichment and long-term secretion profile Clin Hemorheol Microcirc 2010 46 2–3 217 223 21135497 10.3233/CH-2010-1348

[B21] Paulsson JM Held C Jacobson SH Lundahl J In vivo extravasated human monocytes have an altered expression of CD16, HLA-DR, CD86, CD36 and CX(3)CR1 Scand J Immunol 2009 70 4 368 376 10.1111/j.1365-3083.2009.02306.x 19751271

[B22] Bernhagen J Krohn R Lue H Gregory JL Zernecke A Koenen RR Dewor M Georgiev I Schober A Leng L MIF is a noncognate ligand of CXC chemokine receptors in inflammatory and atherogenic cell recruitment Nat Med 2007 13 5 587 596 10.1038/nm1567 17435771

[B23] Solez K Colvin RB Racusen LC Sis B Halloran PF Birk PE Campbell PM Cascalho M Collins AB Demetris AJ Banff '05 Meeting Report: differential diagnosis of chronic allograft injury and elimination of chronic allograft nephropathy (‘CAN’) Am J Transplant 2007 7 3 518 526 10.1111/j.1600-6143.2006.01688.x 17352710

[B24] Merino A Buendia P Martin-Malo A Aljama P Ramirez R Carracedo J Senescent CD14+CD16+ monocytes exhibit proinflammatory and proatherosclerotic activity J Immunol 2011 186 3 1809 1815 10.4049/jimmunol.1001866 21191073

[B25] Vincenti F Current use and future trends in induction therapy Saudi J Kidney Dis Transpl 2005 16 506 513 18202505

[B26] Grüllich C Ziegler C Finke J Rabbit anti T-lymphocyte globulin induces apoptosis in peripheral blood mononuclear cell compartments and leukemia cells, while hematopoetic stem cells are apoptosis resistant Biol Blood Marrow Transpl 2009 15 173 182 10.1016/j.bbmt.2008.11.014 19167677

[B27] Ritter M Buechler C Langmann T Orso E Klucken J Schmitz G The scavenger receptor CD163: regulation, promoter structure and genomic organization Pathobiology 1999 67 5–6 257 261 10725797 10.1159/000028105

[B28] Maniecki MB Etzerodt A Moestrup SK Moller HJ Graversen JH Comparative assessment of the recognition of domain-specific CD163 monoclonal antibodies in human monocytes explains wide discrepancy in reported levels of cellular surface CD163 expression Immunobiology 2011 216 8 882 890 10.1016/j.imbio.2011.02.001 21458881

